# Impregnation of mesenchymal stem cell conditioned media with wortmannin enhanced its antiproliferative effect in breast cancer cells via PI3K/Akt/mTOR pathway

**DOI:** 10.1186/s13104-025-07124-3

**Published:** 2025-03-04

**Authors:** Doha F. Ismail, Mai M. El-Keey, Saad M. Elgendy, Mohamed Hessien

**Affiliations:** 1 Molecular Cell Biology Unit, Division of Biochemistry, Faculty of Science, Tanta University, Tanta, 31527 Egypt; 2 Department of Cancer Biology, National Cancer Institute, Cairo University, Cairo, Egypt

**Keywords:** Breast cancer, MSC conditioned media, PI3K/Akt pathway, mTOR pathway, Wortmannin

## Abstract

**Background/aim:**

Conditioned media derived from Mesenchymal stem cells (MSC-CM) was suggested as a promising alternative cell-free regenerative therapy. It is hypothesized that the synergistic effect of MSC-CM with anticancer drugs may improve their antiproliferative and antimetastatic effects against cancer cells. Herein, the MSC-CM was impregnated with Wortmannin, a pan-PI3K/Akt/mTOR inhibitor, and their combined effect was investigated against breast cancer cells.

**Materials and methods:**

To explore this, the cytotoxic, apoptotic, and autophagic potentials were assessed in luminal-A breast cancer cells (MCF-7).

**Results:**

We found that incubation of MCF-7 to Wort-containing-CM induced apoptosis- and autophagy-mediated cell death, meanwhile prolonged exposure caused massive necrotic cell death. The involvement of MSC-CM effectively reduced Wortmannin IC50 observed in Wort-treated cells. Also, Wort-loaded-CM induced nuclear DNA fragmentation and reduced in vitro cell migration. These findings were associated with a Wort-dependent reduction in cell viability, the formation of the phosphorylated Akt and mTOR proteins, reduced the expression of mRNA, and downregulated the expression of the catalytic domain of phosphatidylinositol-4,5-bisphosphate 3-kinase (PI3K-Ca).

**Conclusion:**

These findings revealed the promising antiproliferative and antimetastasis effects of combining pan-PI3K/Akt/mTOR inhibitors with MSC-derived-CM in breast cancer via the downregulation of PI3K/AKT/mTOR signaling pathways. Further studies are required to validate this chem-regenerative strategy in cancer treatment.

## Introduction

Since their first isolation, five decades ago, by Friedenstein and his coworkers [1], the transplantation of Mesenchymal stem cells (MSCs) has been widely accepted as a promising cell-based therapy in regenerative medicine. However, there is a consensus agreement about the complications associated with MSC engraftment, including quick cell death, miss-homing, hypoxia, and host-related inflammatory responses [2]. Alternatively, preconditioning of MSCs was repeatedly tried to enhance their in vivo survival, proliferation, and therapeutic potential. In this regard, many pharmacological drugs and physical means were employed in preclinical studies before MSC transplantation in animal models [3, 4]. Furthermore, the paracrine role of MSCs was strongly suggested as the next generation of regenerative medicine, where MSC-derived secretome and extracellular microvesicles (EVs) were employed in cell-free preclinical investigations. This was attributed to the plethora of biologically active molecules released by MSCs including cytokines, chemokines, growth factors, antioxidants, and miRNA [5, 6]. These bioactive molecules predispose a wide-range of therapeutic roles like anticancer, antioxidant, antidiabetic, and anti-inflammatory [7–10]. In cancer treatment, for example, many reports have demonstrated the antiproliferative and anti-metastasis effect of MSC-derived conditioned media against many types of cancers like breast cancer [11, 12], squamous cell carcinoma [13], glioma cells [14], urinary tract cancer [15], and lung cancer [16]. These findings were explained by the modulatory effect of MSC-secretome on various cellular targets and signaling pathways involved in cancer initiation and progression [17]. In breast and other cancers, PI3K/Akt signaling acts as a pro-survival mechanism, where it is frequently mutated particularly in genes encoding the PI3K catalytic and regulatory subunits [18]. Moreover, activation of the phosphatidylinositol 3-kinase inhibitor (PI3K/Akt) pathway is associated with drug resistance in breast cancer cells [19]. As the mammalian target of the rapamycin (mTOR) pathway plays a complementary role to PI3K/Akt, a long list of small molecules was presented as common PI3K/Akt/mTOR inhibitors, where many of these compounds are currently used in clinical practice, or still in the clinical development. However, their unsatisfactory chemotherapeutic performance is attributed to the associated adverse [20], immunomodulatory, or pleiotropic effects on tumor angiogenesis [21]. Furthermore, Wortmannin, a PI3K inhibitor extensively used in signaling studies and presented as a potential antineoplastic agent, is not detected in mouse plasma after intravenous administration due to its metabolic conversion to 17-OH-wortmannin. This metabolite is a 10-fold more potent inhibitor of PI3K than Wortmannin [22]. This may explain the associated complications of its role as an anticancer drug. This necessitates the utilization of new therapeutic or delivery modalities, such as nanoformulation or combining them with other bioactive ingredients like MSC-conditioned media. Although MSCs have recently been demonstrated as promising drug delivery vectors for cancer treatment, co-delivery of MSC-conditioned media with anticancer drugs may present a new therapeutic approach to minimize the high dose-related effects. Accordingly, this work was designated to impregnate MSC conditioned media with a common PI3K/Akt/mTOR inhibitor, Wortmannin, to explore their combined antiproliferative effect against luminal-A breast cancer cells and to monitor their impact on PI3l/mTOR pathway.

## Materials and methods

### Key chemicals

Wortmannin (Wort) was supplied by Toronto Research Chemicals, Canada (Cat. no. W499400). Minimum Essential Medium- alpha (MEMα) with nucleosides, Dolbecco’s Minimum Essential Medium (DMEM) with L-glutamine, Penicillin/streptomycin, fetal bovine serum (FBS), and trypsin-ethylenediamine tetra-acetic acid (Trypsin/EDTA) were from Lonza Pharma&Biotech, Switzerland. P-Akt, P-mTOR, and β-actin monoclonal antibodies were from Cell Signaling Technologies, Ma, USA. MSC surface markers antibodies against CD105, CD90, CD45, CD34, and CD19 were from R&D Systems Inc. USA. Acridine orange (AO) and ethidium bromide (EB) were from Sigma.

### Isolation of BM-MSCs and preparation of conditioned media

The study protocol was approved by the Ethical Committee, Faculty of Science, Tanta University (ECL: IACUC-SCI-TU-0214). Bone marrow-derived MSCs were isolated and passaged as previously described [23]. Briefly, bone marrow was aspirated from a male Lewis rat (250 g) by flushing the femoral and tibia bone cavities with PBS in MEMα complete media, containing 10% FBS, and 1% penicillin/streptomycin. After 24 h incubation at 37 °C, and 5% CO_2_, the old media was replaced with fresh complete media, and MSCs were passaged to the 4th passage. MSC phenotypical markers were assessed by flow cytometry to evaluate the expression of MSC-characteristic clusters of differentiation, including CD105 and CD90, and the hematopoietic markers (CD45, CD34, and CD19). Briefly, cells (10^6^ cells/ml) were incubated for 30 min at 4 °C with mouse monoclonal antibodies against different CD markers. The labeled cells were analyzed by a FACSscan flow cytometer (Becton-Dickinson, Franklin Lakes, NJ, USA) using CELLQuest Pro software (Becton-Dickinson). Well-authenticated and viable cells were used to prepare the conditioned media, where cells were incubated in serum-free media for 48 h, after which media were collected and centrifuged to remove any cell derbies.

### Cancer cell lines, culture, and treatment

The breast cancer cell line Michigan Cancer Foundation-7 (MCF-7) was obtained from VACSERA, Cairo, Egypt. Cells were maintained in DMEM with L-glutamine, supplemented with 10% heat-inactivated FBS and 1% Penicillin/Streptomycin. Cells were incubated in humidified conditions at 95% air, 5% CO_2_, and 37 °C. Initially, MCF-7 cells were seeded with low cell density, subcultured with different cell densities, according to the experimental settings, and then treated with Wort or Wort-containing conditioned media.

### Viability assay by sulforhodamine B assay

After cell incubation with Wort or Wort-containing MSC-CM, the sulforhodamine B (SRB) assay was used to measure cellular protein content [24]. Briefly, cells were cultured in DMEM at a density of 2 × 10^4^ cells/well in a 96-well plate. After overnight incubation, for cell attachment, old media were replaced with fresh ones containing different concentrations of Wort and incubated at 37 °C in 5% CO_2_ for 48 h or incubated in Wort-containing CM for 24 h. Next, old media were decanted, cells were fixed with 10% (wt/vol) trichloroacetic acid, stained for 30 min with SRB, washed with 1% (vol/vol) acetic acid, and the protein-bound dye was dissolved in 10 mM Tris base and wells absorbance was measured at 510 nm.

### Apoptosis and autophagy assessments

The apoptosis assay was performed using an Annexin-V FITC kit (Miltenyi Biotec, CA, USA) following the manufacturer’s guidelines. Briefly, MCF-7 cells were cultured in T25 flasks containing complete media. After overnight incubation, cells were treated with Wort or Wort-containing CM for 24–72 h, after which cells were collected by Trypsin/EDTA and then centrifuged at 1000 rpm for 5 min. The cell pellet was resuspended in PBS and incubated with 0.25 µg/ml Annexin V in 1X binding buffer for 15 min, followed by two washes with Wash Buffer. Cells were resuspended again in a binding buffer containing 7-amino-actinomycin (7-AAD) and then analyzed by flow cytometry. In parallel, macroautophagy was assessed by measuring the cellular level of the autophagy marker (LC3II) by fluorescent antibody labeling of the microtubule-associated protein, using Rabbit anti-Homo sapiens MAP1LC3B Polyclonal antibody (MAP1LC3B Antibody, FITC conjugated) (CUSABIO, USA).

### Acridine orange/ethidium bromide dual staining

To assess the rate of cell death and the associated apoptotic morphology acridine orange/ethidium bromide (AO/EB) dual staining was conducted. The assay was performed by staining cells (4 × 10^6^ cells/ml) with 5 µl of AO/EB prepared in PBS. After 10 min incubation at 37 ^o^C, cells were examined under a fluorescent microscope (Zoe, Bio-RAD), where the nuclei of cells with compromised membranes were stained orange-red with ethidium bromide due to its interaction with the fragmented genomic DNA.

### Wound healing assay

To investigate the effect of MSC-CM on cells in vitro migration, wound healing assay was performed. Briefly, after cells were cultured in a 12-well plate and left to subconfluency. A cell-free area (scratch) was made, and the cell monolayer was washed twice with PBS. After incubating cells in Wort-containing DMEM, or Wort-containing CM in serum-free conditions for 48 h, the clear area was estimated by Image J.

### Immunoblotting of akt and mTOR proteins

The ready Prep™ protein extraction kit was used to extract cell protein following the manufacturer’s guidelines. Bradford assay was used to determine protein concentration. For blotting, 20 µg protein was mixed with an equal volume of 2x Laemmli sample buffer (4% SDS, 10% 2-mercaptoethanol, 20% glycerol, 0.004% bromophenol blue, and 0.125 M Tris-HCl, pH 6.8), where the mixture was boiled at 95 °C for 5 min before gel loading. After electrophoresis and membrane blocking at room temperature for 1 h, Primary antibodies of p-Akt and mTOR were diluted in TBST buffer and incubated overnight with each antibody at 4 °C. The blot was washed 4–5 times with TBST buffer and then incubated with the HRP-conjugated secondary antibody (Goat anti-rabbit IgG- HRP-1 mg Goat mab-Novus Biologicals) for 1 h. After another washing step, with TBST, the chemiluminescent substrate was applied, and the signals were captured and analyzed.

### RNA isolation, cDNA synthesis and expression analysis

Quantitative real-time PCR was used to determine the expression of the PI3KCa, Akt, and mTOR genes at the mRNA levels. Initially, total RNA was isolated using GeneJET RNA kit, (ThermoFisher Scientific), and its concentration and quality were assessed. Next, 200 ng RNA was used as a template for cDNA synthesis, using SensiFAST™ cDNA Synthesis Kit (Bioline Inc, USA), following the manufacturer’s guidelines. For RT-PCR quantitation, 50 ng/µl (2 µl) of cDNA was used as a template in 20 µl reactions containing 50 nmol/µl (2 µl) of the genes-specific primers (Table 1), the master mix of fluorescent dye SYBR green 1 (Qiagen) and Hot Star *Taq* DNA polymerase. Reactions were subjected to a thermal cycling program consisting of a single denaturation step followed by 45 cycles (each consisted of a denaturation step at 94 °C for 5 s, annealing at 62 °C, 55 °C and 62 °C and 58 °C, 56.8 °C and 57.9 °C (for PI3Kca, Akt, and mTOR, respectively) and an extension step at 72 °C for 20 s. Reactions were terminated with a single step at 99 °C to produce melt curves. In parallel, the expression of the β actin was used as an internal control to determine the relative quantification of the targeted genes. The critical threshold (C_t_) of target genes was normalized with quantities (Ct) of GAPDH using the 2^−ΔΔCt^.

**Table 1:** Sequence of primers used in the expression analysis

Primer	Sequence (5′-3′)
P-AKT	F: TTC TGC AGC TAT GCG CAA TGT G R: TGG CCA GCA TAC CAT AGT GAG GTT
P-mTOR	F: GCT TGA TTT GGT TCC CAG GAC AGT R: GTG CTG AGT TTG CTG TAC CCA TGT
PI3K-CA	F: GGT TGT CTG TCA ATC GGT GAC TGT R: GAA CTG CAG TGC ACC TTT CAA GC
β-actin	F: AAG ATC CTG ACC GAG CGT GG R: CAG CAC TGT TTG GCA TAG AGG

F: Forward; R: Reverse

**Table 2A:** Candidate cellular targets of Wort as predicted by Similarity Ensemble Approach (SEA, http://sea.bkslab.org/)

Target name	Description	*P* value	Max TC*
PIK3CA	Phosphatidylinositol 4,5-bisphosphate 3-kinase catalytic subunit alpha isoform	6.449e-10	1.00
MYLK	Myosin light chain kinase, smooth muscle	1.318e-07	1.00
MTOR	Serine/threonine-protein kinase mTOR	3.315e-07	1.00
PIK3R1	Phosphatidylinositol 3-kinase regulatory subunit alpha	6.808e-05	1.00
PLK3	Serine/threonine-protein kinase PLK3	0.001813	1.00
PRKDC	DNA-dependent protein kinase catalytic subunit	0.01538	1.00
PLK1	Serine/threonine-protein kinase PLK1	0.08118	1.00
PIK3CB	Phosphatidylinositol 4,5-bisphosphate 3-kinase catalytic subunit beta isoform	0.388	1.00
PIK3CG	Phosphatidylinositol 4,5-bisphosphate 3-kinase catalytic subunit gamma isoform	0.5107	1.00
HSP90AA1	Heat shock protein HSP 90-alpha	0.6738	1.00
PIK3CD	Phosphatidylinositol 4,5-bisphosphate 3-kinase catalytic subunit delta isoform	0.8021	1.00
OPRK1	Kappa-type opioid receptor	1.055e-14	0.34

(*) TC: Tanimoto coefficient

**Table 2B:** Candidate cellular targets of Wort as predicted by Similarity Ensemble Approach TargetNet (http://targetnet.scbdd.com/)

Uniport ID	Protein	Probability
P11511	Aromatase	1.0
P16050	Arachidonate 15-lipoxygenase	1.0
P42336	Phosphatidylinositol 4,5-bisphosphate 3-kinase catalytic subunit alpha isoform	1.0
P42345	Serine/threonine-protein kinase mTOR	1.0
O43353	Receptor-interacting serine/threonine-protein kinase 2	0.975
P07900	Heat shock protein HSP 90-alpha	0.975
P30305	M-phase inducer phosphatase 2	0.975
P08173	Muscarinic acetylcholine receptor M4	0.939
Q00G26	Perilipin-5	0.925
P41235	Hepatocyte nuclear factor 4-alpha	0.902
P28566	5-hydroxytryptamine receptor 1E	0.877
P55211	Caspase-9	0.829
P04058	Acetylcholinesterase	0.822
O60240	Perilipin-1	0.763
P37058	Testosterone 17-beta-dehydrogenase 3	0.762
P04150	Glucocorticoid receptor	0.728
P31941	DNA dC-> dU-editing enzyme APOBEC-3 A	0.491
P29477	Nitric oxide synthase, inducible	0.408

### Computational prediction and statistical analysis

The candidate cellular targets of Wort were predicted by the Similarity Ensemble Approach (SEA) (https://sea.bkslab.org/) and TargetNet (http://targetnet.scbdd.com). Swiss Target Prediction (https://www.swisstargetprediction.ch) was also employed to define cellular regulatory proteins affected by Wort. Data analysis was performed using the SPSS.26.0 software package (IBM, Chicago, IL, USA). All data are presented as means of at least 3 experiments (± standard deviation). The statistical differences among means were tested by ANOVA and post hoc Tukey’s honestly significant difference test. Differences were considered significant at *p* < 0.05. Graphing was performed by Microsoft Excel, and illustrations were performed by BioRender (WWW.BioRender.com).

## Results

### Isolation and phenotypical assessment of BM-MSCs

**Fig. 1:**
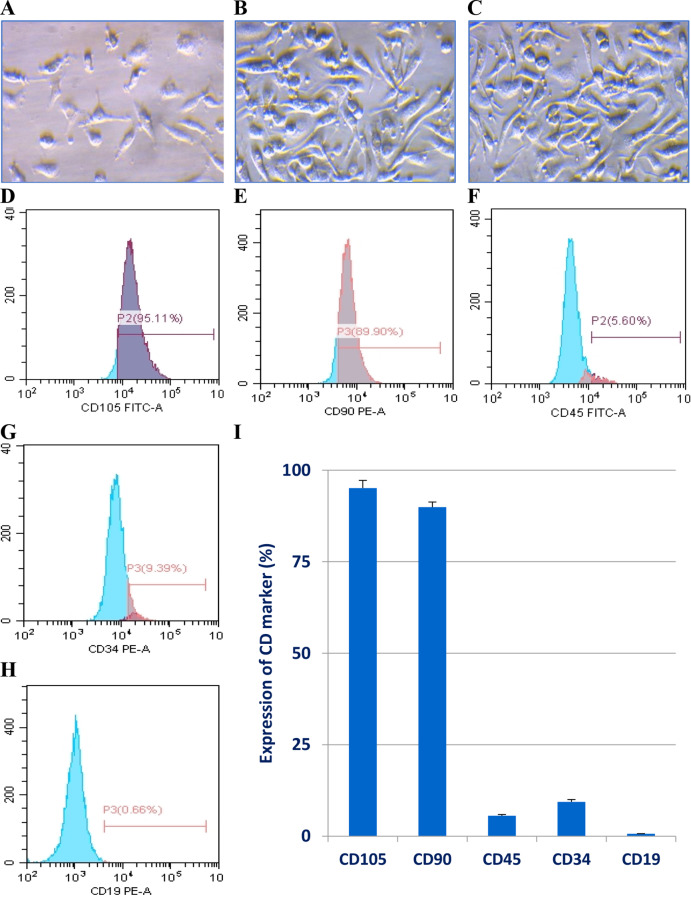
Morphological and phonotypical characterization of BM-MSCs utilized to obtain MSC-CM. **A**-**C** are representative micrographs of the BM-MSCs at day 4, day 12, and 4th passage cells. Adherent cells exhibited a typical fibroblast-like spindle shape. “1D” through “1H” represent the expression of the clusters of differentiation (CD), including positive markers (CD105&CD90) and three hematopoietic markers (CD45, CD34, and CD19). The bar graph “1I” depicts the mean (± SD) of the relative expression of 3 independent flow cytometry analyses for each marker

Initially, BM-MSCs were isolated from the rat’s bone, maintained, and propagated to the 4th passage. Cells were phenotypically authenticated by assessing the expression levels of both the mesenchymal and hematopoietic surface markers. Figure 1A and C demonstrates that MSCs demonstrated a typical spindle fibroblast-like shape under a phase contrast microscope. Also, they highly expressed the mesenchymal-specific markers (CD105 and CD90) (Fig. 1D and E) and minimally expressed the hematopoietic markers including CD45, CD34, and CD19 (Fig. 1F and H). The averages of CD expression are shown in Fig. 1I. Well-authenticated BM-MSCs were grown in serum-free media and the conditioned media were collected and used either alone (Wort-free MSC-CM) or enriched with 50, 100, or 250 nM Wort to treat MCF-7 cells.

### Cytotoxic effect of wortmannin-enriched MSC-conditioned media on MCF-7 cells

**Fig. 2:**
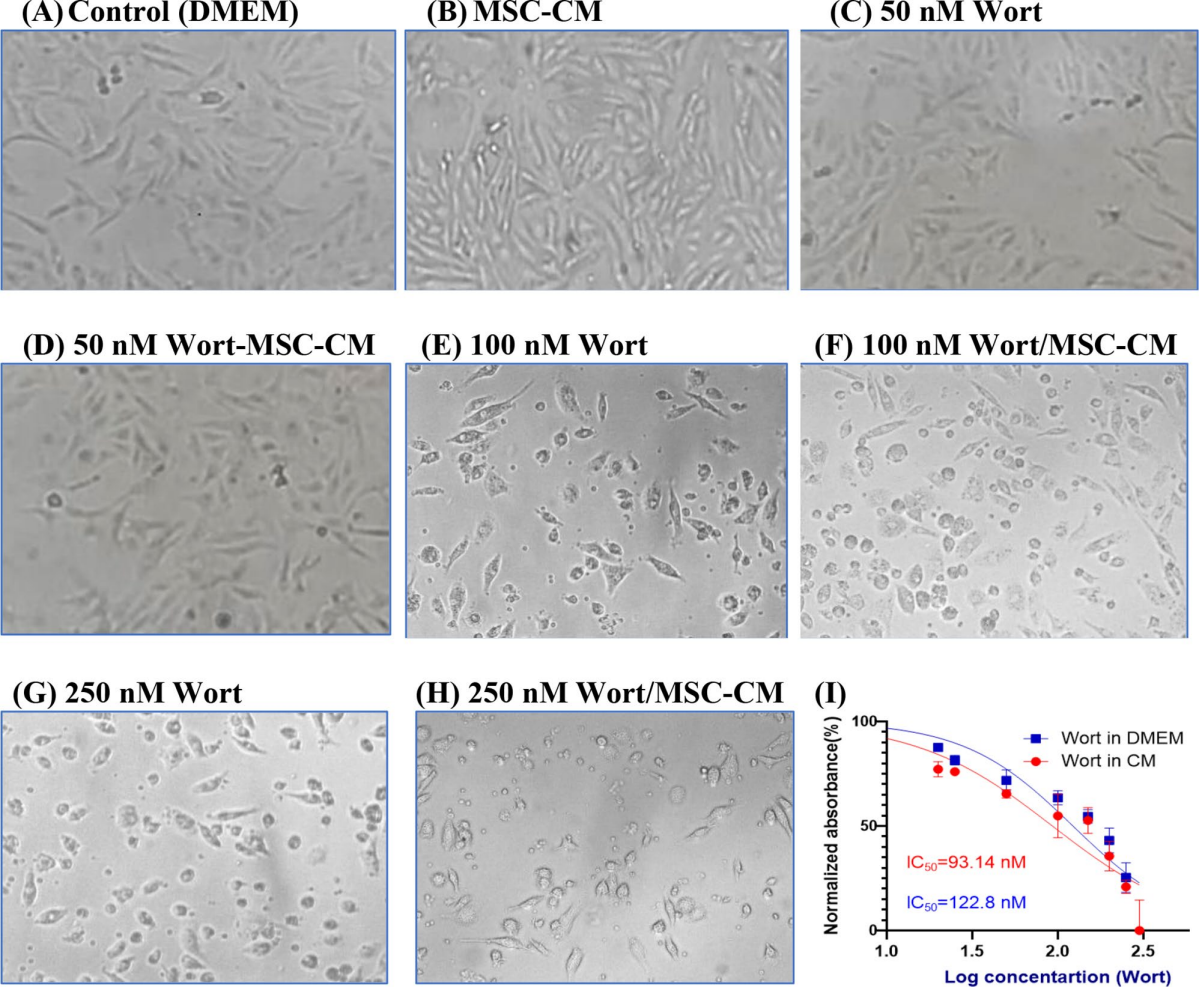
Cytotoxic effect of Wort and Wort-loaded CM on MCF-7 cell morphology and viability. 2 A, through 2 F are representative phase-contrast micrographs of untreated MCF-7 cells (2** A**), cells grown in MSC-CM (2**B**), in 50 nM Wort (2**C**), in CM-containing 50 nM Wort (2**D**), 100 nM Wort (2**E**), MSC-CM containing 100 nM (2** F**), 250 nM Wort (2**G**), or 250 nM Wort-containing secretome (2**H**). 2I demonstrates the normalized changes in cell viability, assessed by SRB assay, after cells were treated with different concentrations of Wort (blue), or CM-containing different concentrations of Wort (Red). Results are expressed as mean (± SD) from 4–6 independent measurements

**Fig. 3:**
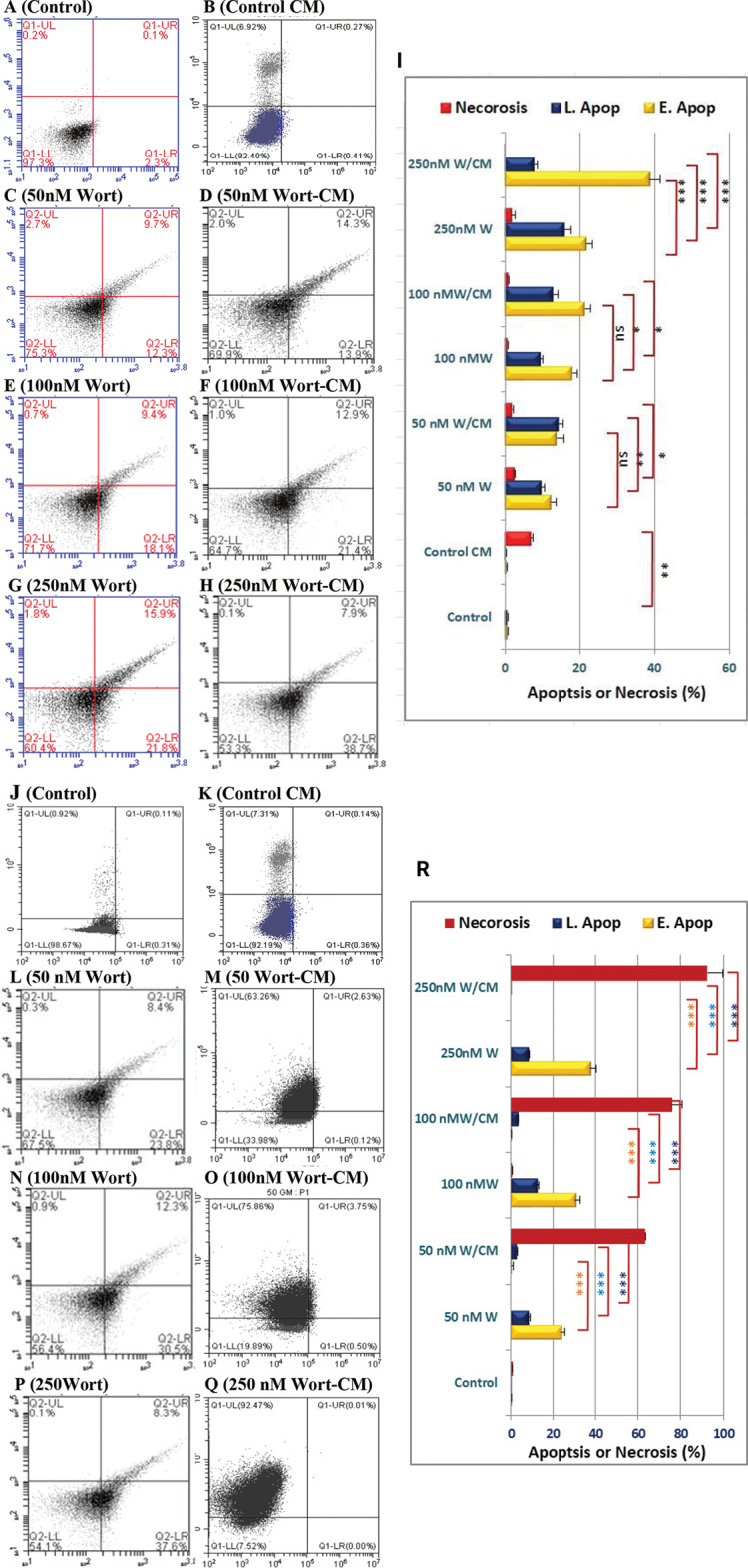
Detection of apoptosis-mediated cell death in MCF-7 cells after incubation with Wort or Wort-loaded CM. Panels “I” and “II” include scatter blots of cells incubated for 24–72 h, respectively. Cells were dually stained with Annexin-V/7AAD and subjected to flow cytometry. In each blot, the X and Y axes represent Annexin V and 7AAD, respectively. Viable and early apoptotic cells are represented in the lower left and right quadrants, respectively. Cells in the late apoptosis or dead cells are represented in the upper right and left quadrants, respectively. Types of treatments are shown above each panel. Bar graphs “3H” and “3R” represent the comparative analysis between Wort treatment and the corresponding Wort-containing CM. Abbreviations: Wort: Wortmannin and S: secretome. (***): *P* < 0.001, (**): *P* < 0.01, (*): *P* < 0.05 and ns = non-significant. E. Apop and L. Apop refer to early and late apoptosis, respectively

Exposure of MCF-7 cells to Wort and Wort-containing CM, for 24 h, progressively led to cytotoxicity, where they demonstrated lower cell density and apoptotic morphological features including cell rounding, shrinkage, and plate detachment suggesting the development of apoptosis (Fig. 2C-H), compared to the untreated cells (Fig. 2A) or cell treated incubated in Wort-free conditioned media (Fig. 2B). The cytotoxic effect, assessed by SRB, revealed a Wort-dependent reduction in cell viability and a progressive increase in cell death when MCF-7 cells were grown in media containing increasing concentrations of Wort (Fig. 2.I). The IC_50_ concentration of Wort-loaded-CM was lower than the corresponding value in cells treated with Wort signifying the increased cell sensitivity to Wort-loaded MSC-CM. Annexin V/7AAD dual staining, followed by flow cytometry, demonstrated that MCF-7 cells treated, for 24 h, with Wort alone showed significant apoptosis (Fig. 3I. C, E, and G). Furthermore, more apoptotic effects were observed when cells were incubated in the corresponding Wort-containing CM (Fig. 3.I. D, F, and H). Prolonged incubation of cells, for 72 h, in 50 nM, 100 nM, or 250 nM Wort-containing-CM led to massive cell death in 63.26%, 75.86%, and 92.47% of cells, respectively (Fig. 3. II).

### Apoptosis detection by fluorescent microscopy

**Fig. 4:**
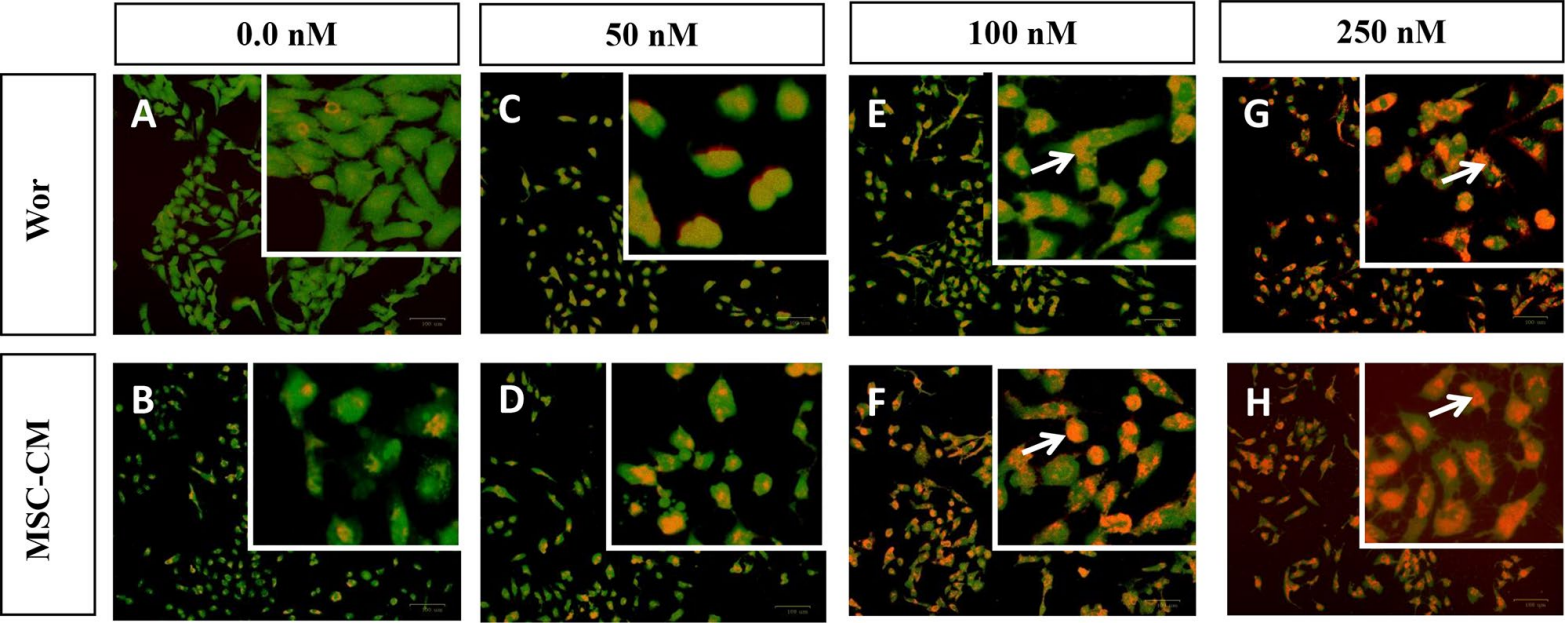
Assessment of apoptosis by acridine orange/ethidium bromide (AO/EB) dual staining. MCF-7 cells were untreated (4**A**), treated with 50 nM (4**C**), 100 nM (4**E**) or 250 nM (4**G**) Wort or MSC-CM containing similar concentrations (4**B**, 4**D**, 4**F**, and 4**H**, respectively). Adherent cells were washed and stained with AO/EB. White arrows refer to nuclear red-orange staining with ethidium bromide, indicating the compromised cell membranes and genomic DNA fragmentation

To investigate the impact of treatments on the cell membrane and nuclear integrity, dual staining with acridine orange/ethidium bromide was performed (Fig. 4A and H). In comparison to untreated cells or cells treated with low doses of Wort (Fig. 4A and C, respectively), higher concentrations (100 or 250 nM) resulted in nuclei exhibiting dark orange-red fluorescence. This effect is attributed to the disruption of cell membranes, allowing ethidium bromide to interact with single-stranded (fragmented) DNA (Fig. 4E and G, respectively). Furthermore, treatment with Wort-containing CM enhanced the lethal effect as cells demonstrated more intense red staining (Fig. 4F, and 4H) indicating the compromised cell membrane and the development of apoptosis or necrosis.

### Autophagy assessments

**Fig. 5:**
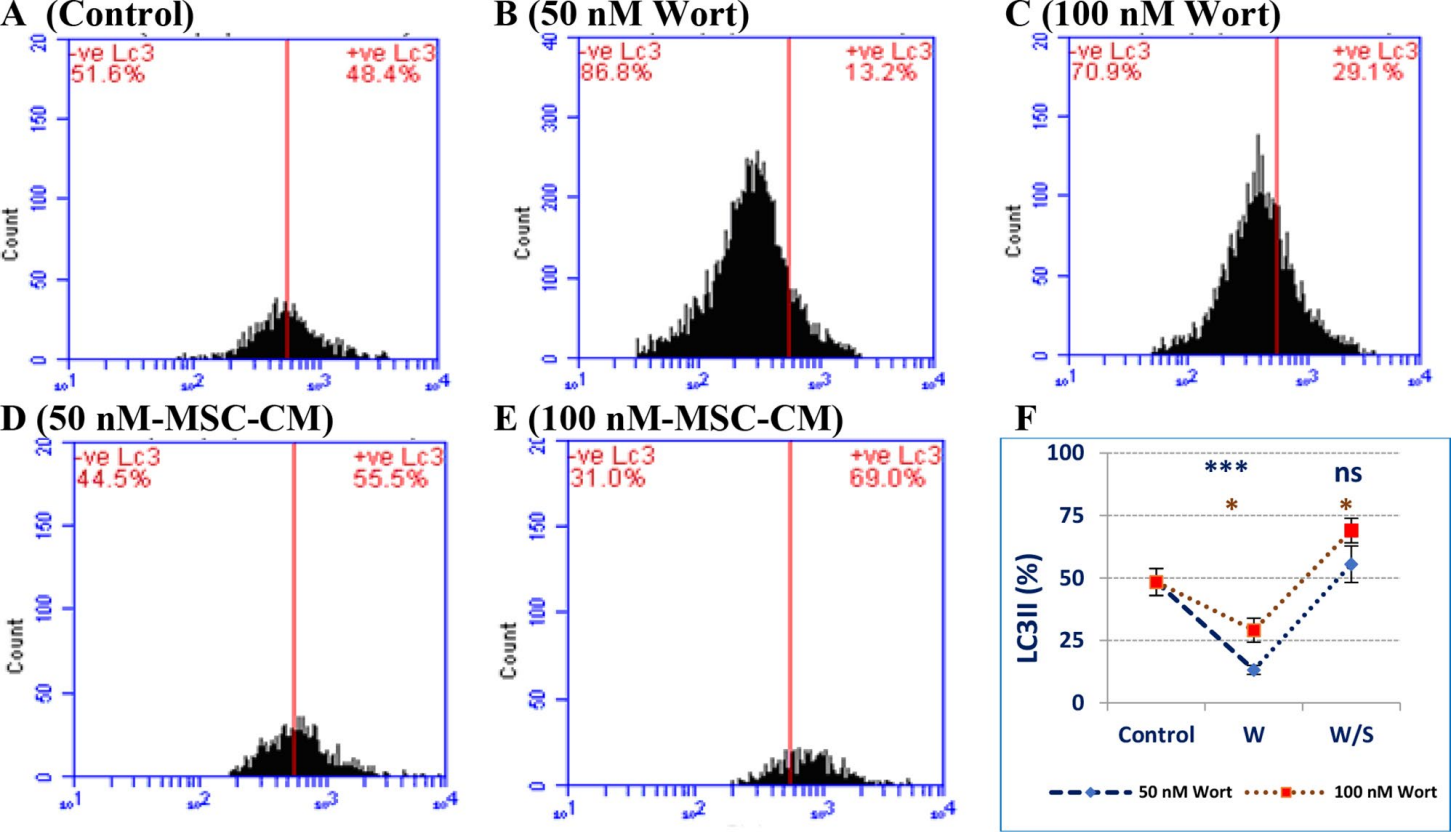
Induction of autophagy in MCF-7 cells as indicated by the percentage of LC3II protein. Cells were left untreated (5**A**), treated with, 50 nM (5**B**), or 100 nM (5**C**) Wort alone, or similar concentrations combined with MSC-CM (5**D**, 5**E**). “F” represents the relative changes in the level of the autophagy marker. Abbreviations: W: Wortmannin, S: MSC-CM, (***): *P* < 0.001, (*): *P* < 0.05, relative to the untreated cells

Next, the autophagy-mediated cell death was assessed by measuring the LC3II protein. We observed that Wort significantly reduced the level of LC3II protein (Fig. 5C) relative to its basal level found in the control cells (Fig. 5A). However, loading MSC-CM with Wort increased the level of LC3II protein compared to its basal level (untreated cells) or cells individually treated with Wort (Fig. 5D and F).

#### Effect of wortmannin-loaded MSC-secretome on cell metastasis

**Fig. 6:**
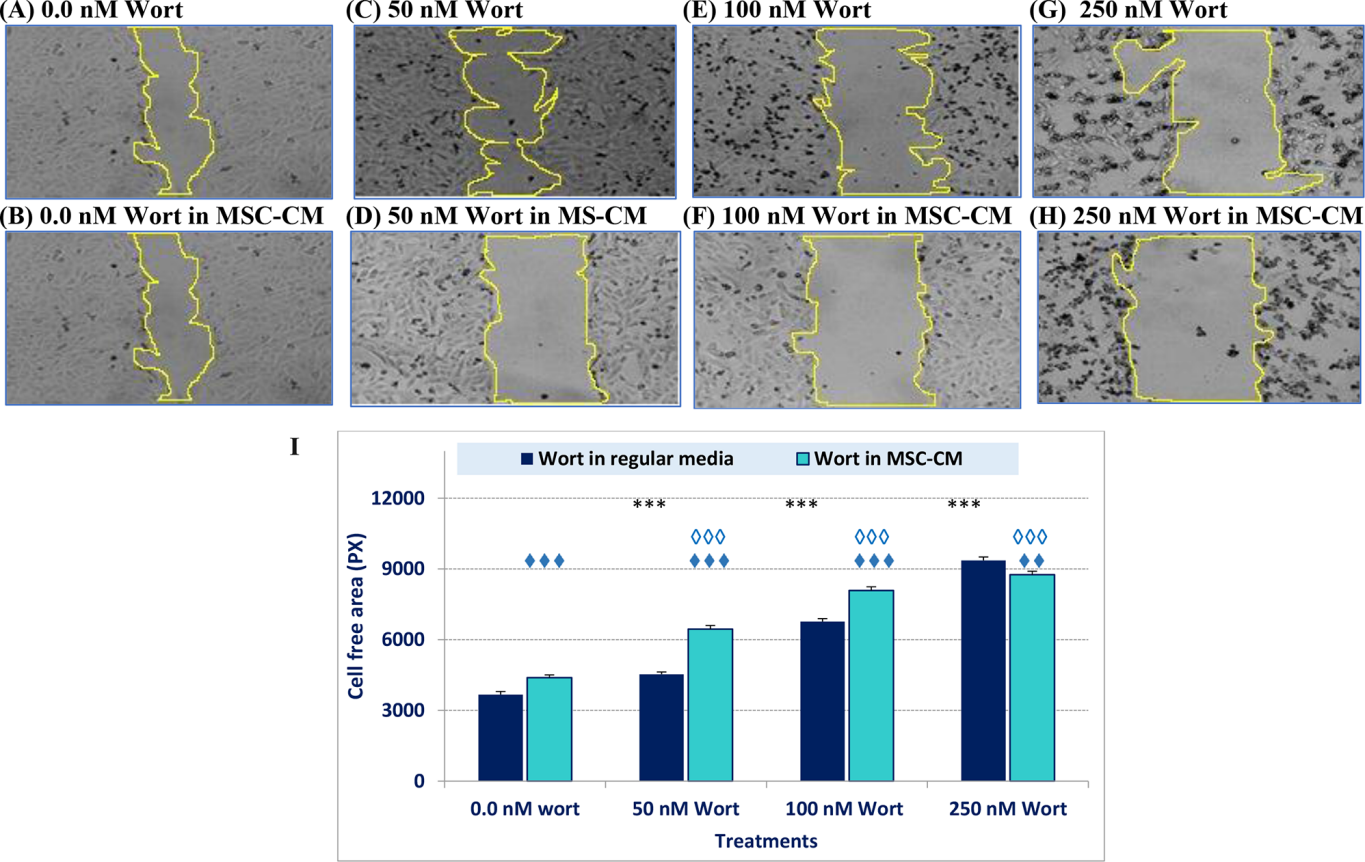
In vitro cell migration assay of MCF-7 cells treated with Wort or the Wort-loaded MSC-CM. “6**A**, 6**C**, 6**E**, and 6G are representative micrographs of cells treated with 0.0, 50, 100, or 250 nM Wort in regular media (DMEM). Panels 6**B**, 6**D**, 6**F**, and 6H are representative cell migration images of cells treated with Wort-containing CM. The bar graph “6I” shows the changes in the wound area assessed by Image J. Abbreviations: CM: Conditioned media, Wort: Wortmannin. (♦♦♦) and (***) refer to P < 0.001 between the indicated treatments versus the corresponding control treatment. (◊◊◊) refers to a significant difference (P < 0.001) between Wort and Wort containing CM

To further explore the impacts of wort-loaded MSC-CM on cell migration, Wound healing assay was performed after cells had been treated with Wort or Wort-containing MSC-CM (Fig. 6). Different concentrations of Wort (Fig. 6C and E, and 6G) and Wort-containing CM (Fig. 6.D, 6 F, and 6 H) suppressed cell migration compared to the corresponding controls (Fig. 6A and B, respectively). The area changes are depicted in Fig. 6I.

#### Impact of wortmannin containing MSC-secretome PI3K/Akt-mTOR pathway

**Fig. 7:**
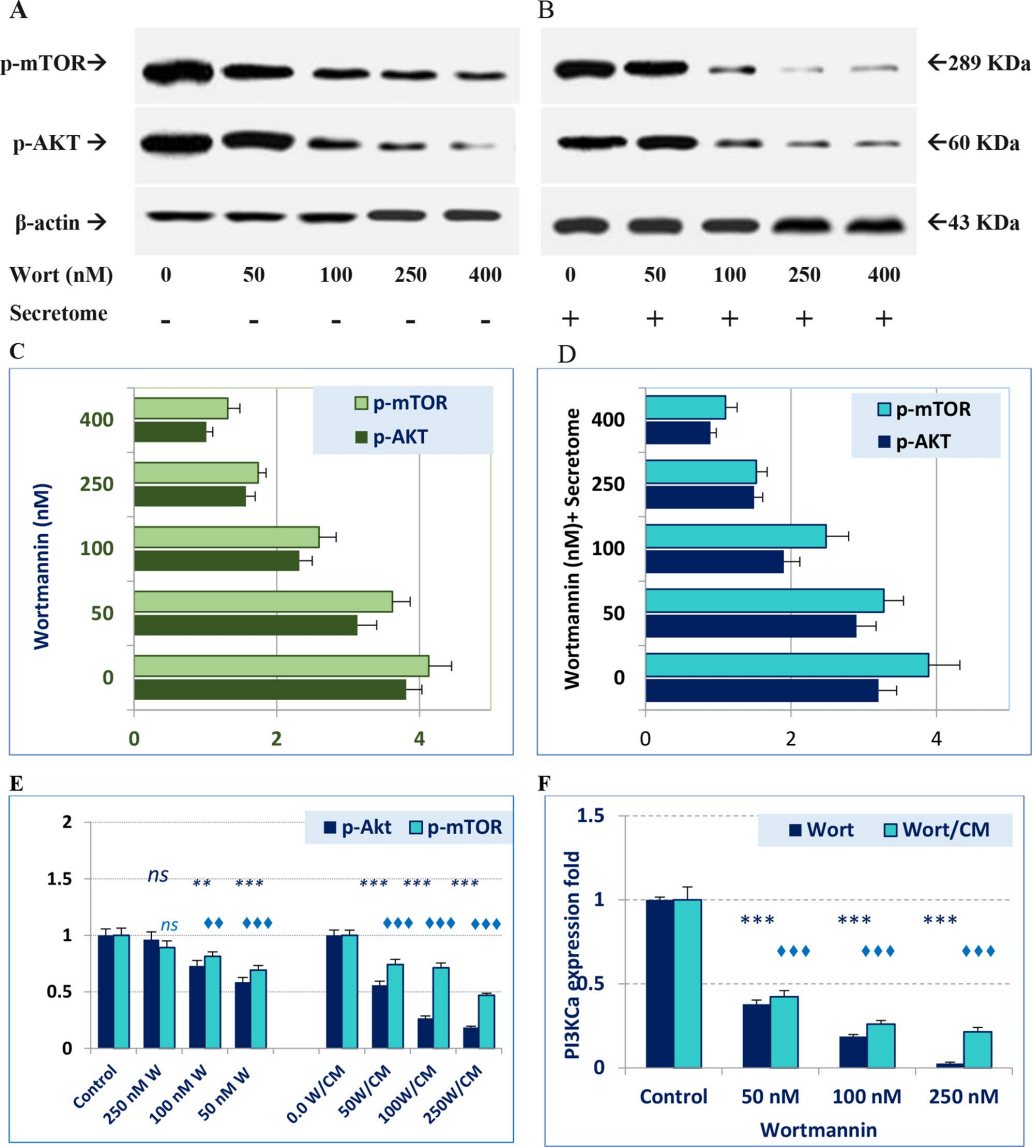
Immunoblot and expression analysis of the Akt, mTOR, and PI3K-Ca of MCF-7 cells in response to Wort, or Wort-loaded MSC-CM. Phosphorylated forms of Akt and mTOR proteins were assessed in cells treated with different concentrations of Wort (7**A**), or Wort-loaded CM (7**B**) progressively reduced relative to the untreated cells and the corresponding β-actin, as a loading control. Both “7**C**” and “7**D**” depict the corresponding band intensities. Expression of P-AKT, m-mTOR at the mRNA level is shown in “7**E**”, whereas “7**F**” demonstrates the stepwise downregulation of PI3K-catalytic subunit (PI3K-Ca)

To study the mechanism of action and the involvement of PI3K/Akt/mTOR pathway in the antiproliferative effect of Wort and MSC-CM was assessed by immunoblotting. Treated cells were lysed and then the expression level of Akt and mTOR and their phosphorylated forms (P-Akt and P-mTOR) were evaluated by immunoblotting (Fig. 7A and D). We found that the formation of the phosphorylated forms gradually decreased as the Wort concentration increased, either alone or combined with MSC-CM. Similarly, the expression of Akt, mTOR, and the catalytic subunit of PI3K (PI3KCa), at the mRNA level, was downregulated (Fig. 7E and F).

## Discussion

**Fig. 8:**
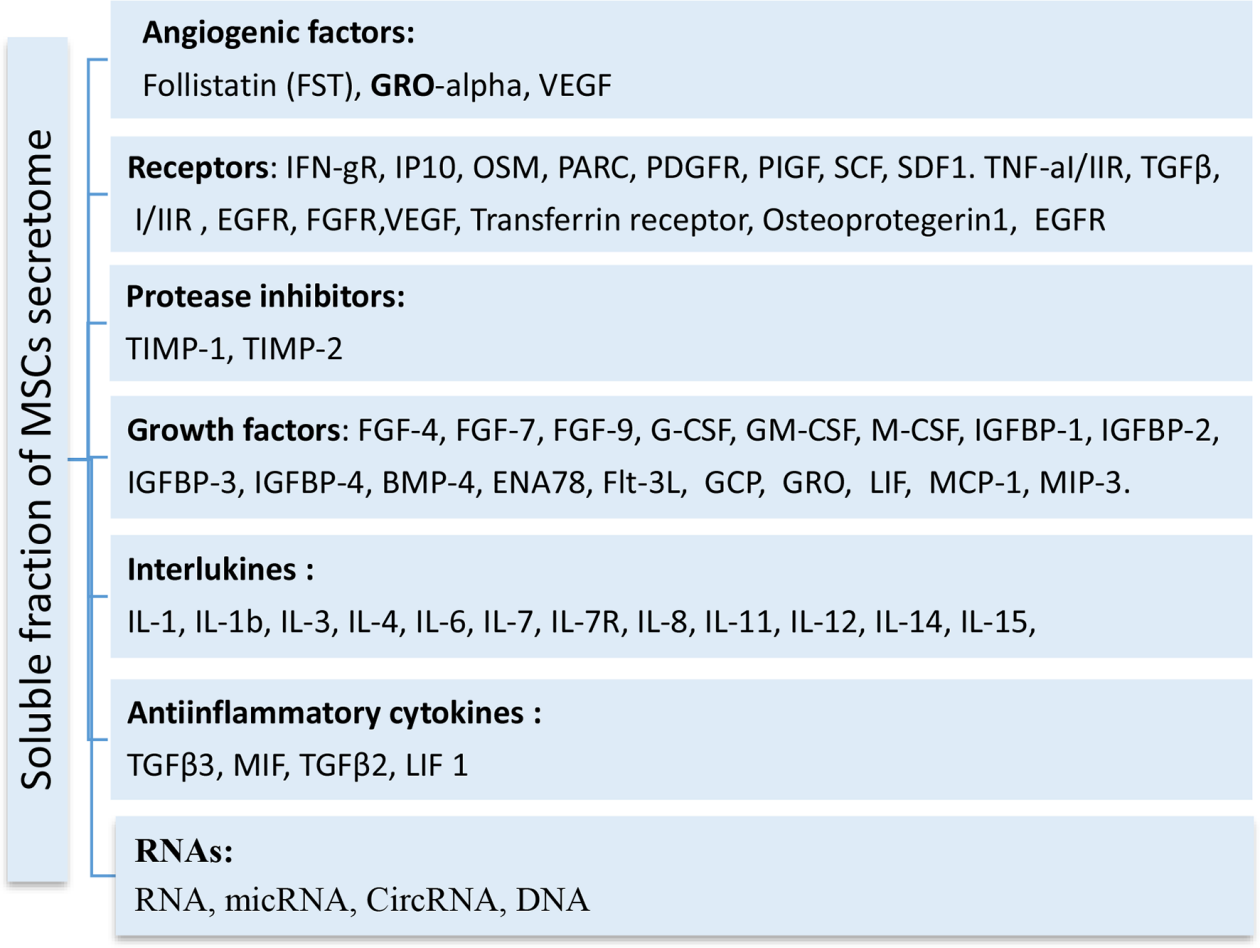
Main secretome components released by MSCs. The secretome includes a mixture of growth factors, angiogenic factors, soluble receptors, and protease inhibitors. Also, it contains immunomodulatory factors including interleukins, anti-inflammatory, and some RNA species (based upon Phinney et al., (2006) [45]

**Fig. 9:**
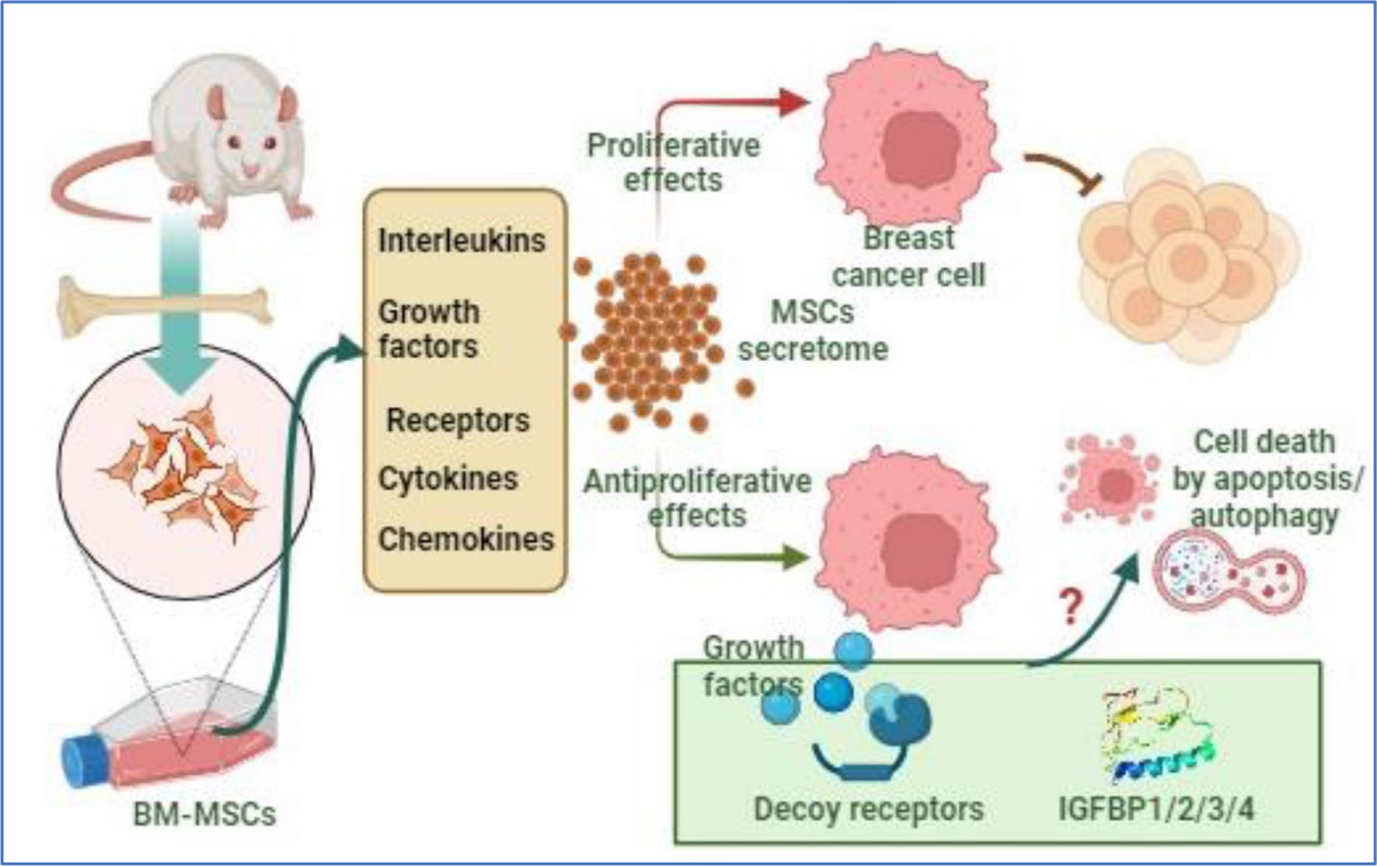
Suggested antiproliferative effect of MSC secretome. MSC secretome contains mixture of regulatory proteins including interleukins, growth factors, cytokines, chemotactants, and soluble receptors. The antiproliferative role of these proteins is suggested through the trapping growth-inducing factors by the decoy receptors or the growth inhibitory effect of some growth factors like insulin-like binding proteins

**Fig. 10:**
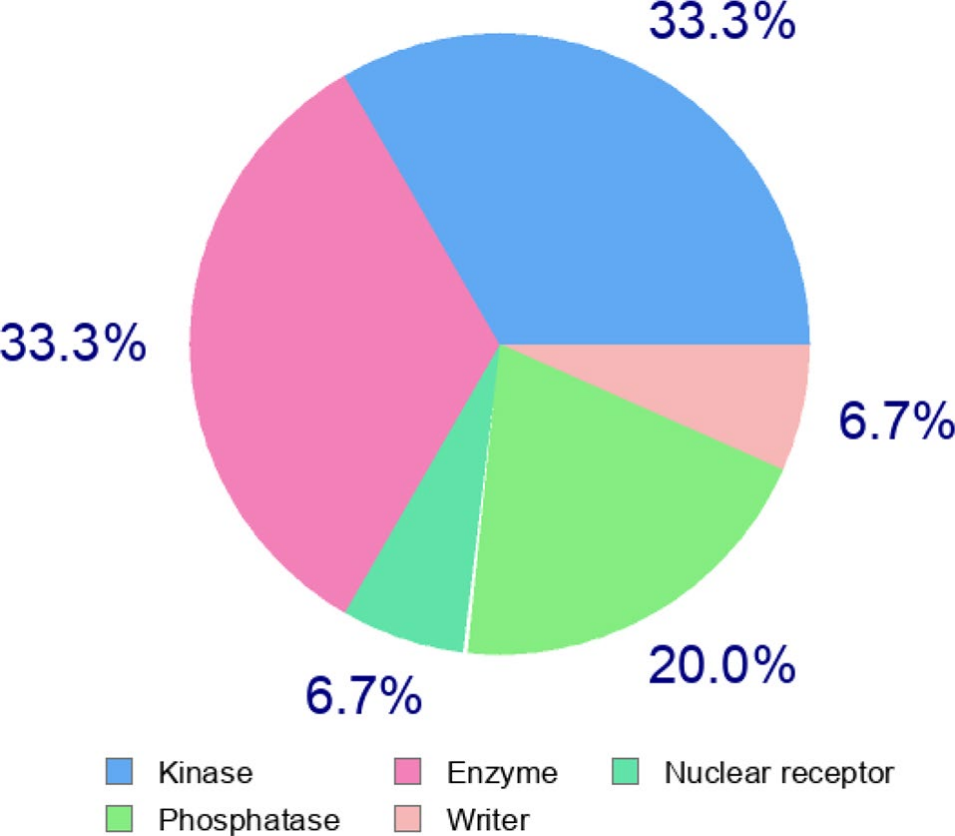
Swiss Target Prediction (https://www.swisstargetprediction.ch) of major classes of regulatory proteins targeted by Wort. The figure indicates that Wort interacts with at least 5 subsets of cellular proteins, mainly including kinases and phosphatases

This work suggested that combining anticancer drugs, like pan PI3K/Akt/mTOR inhibitors, with MSC-CM enhanced its cytotoxic, apoptotic, and autophagy-mediated cell death compared to the individual effect of MSC-CM or Wortmannin. This observation was demonstrated by the increased cell death mediated by apoptosis and autophagy. Moreover, this combination restricted the cell migration in vitro. The limited survivability of the transplanted MSCs, observed in preclinical studies, is likely compensated by their paracrine effect via the biological molecules they release [8]. Also, transfusion of MSC-conditioned media in experimental models bypasses the post-transplantation complications. Also, the easy acquisition, storage, and delivery add more advantages to the CM-mediated therapeutic approach. In this regard, the genome of the MSCs is actively transcribed and translated into many proteins including intracellular, membrane-bound, and excreted proteins [25]. The latter, baseline-secretome, is anticipated as a main deriving force of the healing potential of MSCs due to the cytokines, chemokines, interleukins, and growth factors it contains (categorized in Fig. 8). Although cytokines and interleukins are known to stimulate fibroblast proliferation, they may inhibit the growth of other cell types [26, 27]. Also, some growth factors, like insulin-like growth factor binding proteins (IGFBP1/2/3/4) demonstrate antiproliferative by binding to IGFs and inhibiting their proliferative effect [28, 29]. The inclusion of some soluble decoy receptors in the MSC secretome, like Osteoprotererin1 [30], may limit the proliferative effect of cytokines and growth factors (Fig. 9). These scenarios may explain the antiproliferative effect we observed in MCF-7 when treated with Wort-free conditioned media. To enhance such effect, and minimize the complications associated with chemotherapy, we aimed to impregnate the MSC-CM with an anticancer drug like PI3K/Akt/mTOR inhibitor. This pathway supports the survival of cancer cells and promotes cell metastasis via the modulation of proapoptotic proteins, such as Bad and p53 [31–33], or its endogenous controllers like PETN [34]. This explains why the PI3K/Akt/mTOR pathway is targeted by many drugs, particularly those targeting PI3Ks activity, AKT, and/or mTOR proteins. The drug we used (Wort) is known to inhibit all PI3K classes (IA, IB, II, and III) [35–37], and its direct effect involves their catalytic domains. However, many other cellular targets with high interaction probabilities were predicted (shown in Tables 2A and 2B). Also, there is no evidence of the interaction between Wort and the immunomodulatory factors, where Wort targets enzymes (such as kinases, and phosphatases), and nuclear receptors (Fig. 10). Notably, Wort-loaded-CM increased the cytotoxic effect even with a lower concentration of Wort, where treatment of cells with CM loaded with 50 nM reduced the cell viability to 64% compared to 90% in Wort-treated cells. The cytotoxicity of the Wort-loaded CM was mediated by apoptosis and autophagy-mediated cell death and associated with nuclear changes including chromatin condensation and nuclear fragmentation due to the overproduction of ROS [38]. Unexpectedly, incubation of breast cancer cells with Wort-loaded CM for a longer period led to a massive cytotoxic effect. Such drastic effect was previously reported in MCF-7 following their exposure to cisplatin, and caffeine [39], and could be attributed to the unidentified soluble molecules the secretome includes [40]. In previous studies, MSCs were loaded with paclitaxel (PTX), commonly used in breast cancer treatment, where this combination demonstrated a pronounced inhibitory effect on the survival, migration, and tumorigenicity of the triple-negative breast cancer (TNBC) cells [41]. Furthermore, as PI3K/Akt pathway is the classical upstream signaling pathway of nutrient deprivation-induced autophagy [42]. In agreement with previous reports [43], Wort inhibits autophagy primarily by targeting class III PI3K and blocking the production of PI3P, thereby preventing the initiation and progression of autophagosome formation. MSC-derived CM, in contrast, can influence key autophagy regulators, such as the AMPK/mTOR signaling pathway, and promote the formation of autophagosomes [44]. This explains the restoration of the formation of LC3II observed when cells were treated with Wort Containing conditioned media. Also, it may suggest the PI3K-independent autophagic effect of MSC-CM [45]. The progressive decrease in the phosphorylation of Akt and mTOR may indicate the direct inhibitory effect of both pathways and the development of apoptosis. Although the inhibition of mTOR may occur as a downstream consequence of PI3K/Akt inhibition, some reports suggested that Wort may inhibit mTORC1 by an Akt-independent mechanism and may involve small GTPases like Rheb [46].

## Conclusion

Taken together, this work suggests that the impregnation of MSC-CM presents a promising chemo-regenerative therapeutic strategy against breast cancer. The anti-proliferative effect of Wort-loaded MSC-CM involves both apoptosis and autophagy via Wort-dependent PI3K/Akt/mTOR inhibition. These observations are attributed to the biological molecules released by MSCs, which enhance the cytotoxic effect even when loaded with lower doses of the anticancer drug.

### Limitations

Combining conventional anticancer drugs with MSC secretome may present a new chemo-regenerative therapeutic approach with many advantages compared to traditional chemotherapy or cell-based transplantation because it is less immunogenic and permits the utilization of lower concentrations of the anticancer drug, thus minimizing the associated adverse effects. However, future studies are recommended to include more drugs in different cell lines and animal models. Also, to emphasize this approach, further studies should explore the direct molecular interactions between Wort and similar drugs with different MSC mitogenic proteins like growth and angiogenic factors.

## Data Availability

The authors declare that the data supporting the findings of this study are available within the paper and its supplementary Information files. Should any raw data files be needed in another format they are available from the corresponding author upon reasonable request.
